# Influence of systolic blood pressure trajectory on in-hospital mortality in patients with sepsis

**DOI:** 10.1186/s12879-023-08054-w

**Published:** 2023-02-13

**Authors:** Jia-Liang Zhu, Shi-Qi Yuan, Tao Huang, Lu-Ming Zhang, Xiao-Mei Xu, Hai-Yan Yin, Jian-Rui Wei, Jun Lyu

**Affiliations:** 1 Department of Intensive Care Unit, The First Affiliated Hospital of Jinan University, No.613, Huangpu Road West, Guangzhou, 510630 Guangdong Province China; 2 Guangzhou Women and Children’s Medical Center, Guangzhou Medical University, No. 9 Jinsui Road, Guangzhou, 510630 Guangdong Province China; 3 Department of Neurology, The First Affiliated Hospital of Jinan University, No.613, Huangpu Road West, Guangzhou, 510630 Guangdong Province China; 4 Department of Clinical Research, The First Affiliated Hospital of Jinan University, 613 West Huangpu Avenue, Tianhe District, Guangzhou, 510630 Guangdong Province China

**Keywords:** Latent growth mixture modeling, Systolic blood pressure, In-hospital mortality, MIMIC-III database, Retrospective study

## Abstract

**Background:**

Numerous studies have investigated the mean arterial pressure in patients with sepsis, and many meaningful results have been obtained. However, few studies have measured the systolic blood pressure (SBP) multiple times and established trajectory models for patients with sepsis with different SBP trajectories.

**Methods:**

Data from patients with sepsis were extracted from the Medical Information Mart for Intensive Care-III database for inclusion in a retrospective cohort study. Ten SBP values within 10 h after hospitalization were extracted, and the interval between each SBP value was 1 h. The SBP measured ten times after admission was analyzed using latent growth mixture modeling to construct a trajectory model. The outcome was in-hospital mortality. The survival probability of different trajectory groups was investigated using Kaplan-Meier (K-M) analysis, and the relationship between different SBP trajectories and in-hospital mortality risk was investigated using Cox proportional-hazards regression model.

**Results:**

This study included 3034 patients with sepsis. The median survival time was 67 years (interquartile range: 56–77 years). Seven different SBP trajectories were identified based on model-fit criteria. The in-hospital mortality rates of the patients in trajectory classes 1–7 were 25.5%, 40.5%, 11.8%, 18.3%, 23.5%, 13.8%, and 10.5%, respectively. The K-M analysis indicated that patients in class 2 had the lowest probability of survival. Univariate and multivariate Cox regression analysis indicated that, with class 1 as a reference, patients in class 2 had the highest in-hospital mortality risk (P < 0.001). Subgroup analysis indicated that a nominal interaction occurred between age group and blood pressure trajectory in the in-hospital mortality (P < 0.05).

**Conclusion:**

Maintaining a systolic blood pressure of approximately 140 mmHg in patients with sepsis within 10 h of admission was associated with a lower risk of in-hospital mortality. Analyzing data from multiple measurements and identifying different categories of patient populations with sepsis will help identify the risks among these categories.

**Supplementary Information:**

The online version contains supplementary material available at 10.1186/s12879-023-08054-w.

## Introduction

Sepsis is a life-threatening organ dysfunction caused by a dysregulated host response to acute infection. Sepsis remains the most common cause of mortality in critically ill patients [1]. Early identification of patients at a high risk of sepsis and individualized treatment are therefore effective measures to reduce the mortality risk of patients with sepsis. One study found that a decrease in systolic blood pressure (SBP) within 48 h of admission was associated with increased 180-day cardiovascular mortality in patients with acute heart failure [2]. Another study found that a decrease in SBP during the first day of admission in patients with acute heart failure promoted worsening renal function and was associated with worse prognosis [2]. Another previous study found that patients with acute myocardial infarction and low SBP had a higher risk of adverse events. A Chinese study found that patients with acute myocardial infarction had the lowest 5-year overall and cardiovascular mortality risk when their mean SBP remained between 90 and 130 mmHg during the first week after admission [3].

Previous research suggests that the trend change in SBP after admission has predictive value for the prognosis of cardiovascular-related diseases. However, there is no relevant research on the prognosis of patients with sepsis according to the trajectory of SBP after admission.

The purpose of this study is to identify the SBP trajectory with the best and worst prognosis in patients with sepsis by establishing a Latent growth mixture modeling (LGMM) model. The SBP value of the patients with the best prognosis was used as a maintenance target to reduce the risk of in-hospital mortality in patients with sepsis.

## Methods

### Data sources

The MIMIC-III is a large public database that recorded data on patients in the intensive care unit (ICU) of Beth Israel Deaconess Medical Center between 2008 and 2019, and it contains medical health data and records for more than 40,000 patients. The MIMIC-III database records a large amount of data such as demographic information, laboratory test information, patient medication information, patient imaging reports, and patient admission and discharge information [5]. We completed the course and passed the examination from the National Institutes of Health that authorized us to use the database (certificate number: 45848364).

### Sample selection

The diagnosis of sepsis was according to The Third International Consensus Definitions for Sepsis and Septic Shock (sepsis-3). The exclusion criteria were (1) age < 18 years, (2) blood pressure not measured ten times after admission, or (3) a single hospital stay lasting longer than 90 days.

### Variables and outcome

Variables for this study included basic patient information, vital signs within 24 h of admission, laboratory tests, and comorbidities. Basic patient information included age and sex. Vital signs include respiratory rate, heart rate, SBP, diastolic blood pressure, body temperature, and oxygen saturation. In the MIMIC-III database, SBP was recorded hourly after admission, and we extracted ten SBP values measured chronologically 10 h after admission. Laboratory tests included white blood cell count, neutrophil ratio, platelet count, lymphocyte count, serum sodium, aspartate aminotransferase, albumin, creatine kinase, creatine kinase isoenzyme MB, creatinine, international normalized ratio, and blood glucose. Comorbidities included congestive heart failure, cerebrovascular disease, and chronic pulmonary disease. The outcome of the study was all-cause mortality during hospitalization.

### LGMM

Latent growth mixture modeling (LGMM) has recently been widely used to evaluate longitudinal data. Li [4] used LGMM to study the different trajectories of leptin in children and their relationships with cardiovascular metabolism in adolescence. Muthén [6] proposed a latent growth mixed model in 1999, which is a new analytical method for identifying trends in longitudinal data. The principle of LGMM is to assume that there is heterogeneity in the population; that is, that there are multiple subgroups with different development trajectories. In the present study of patients with sepsis from the Medical Information Mart for Intensive Care-III (MIMIC-III) database, we extracted ten SBP values in the chronological order of data recording, performed trajectory analysis and classification, and studied the hospitalization period of each category of sepsis patients risk of mortality.

First, a primary item model with only one category was established, and the number of latent categories was then increased based on the primary items, and finally the number of trajectory latent categories was determined. The evaluation indicators of the goodness of fit of the model included the Akaike Information Criterion (AIC), the Bayesian Information Criterion (BIC), the sample-size adjusted BIC (SABIC), and the information entropy (Entropy). Karen found that SABIC is the best information indicator [7]. We therefore determined the optimal number of classes according to the principle that a lower SABIC corresponds to a better goodness of fit of the model. Entropy was used to evaluate the classification accuracy of the model, and the value was between 0 and 1. Generally, if the value is higher than 0.8, the model is considered to have high classification accuracy [8]. The average posttest grouping probability also reflected the probability that each individual will be assigned to the corresponding subgroup after the number of categories is determined, and is usually accepted as greater than 70%. To ensure the statistical power of the data analysis, we stipulated that the sample size of each subgroup should not be less than 2% of the total sample size.

### Statistical analysis

Data were extracted from the MIMIC-III database using Navicat Premium 15 with Structured Query Language in PostgreSQL (version 12.0) [9]. Variables for which the proportion of missing values exceeded 10% were excluded, and missing values for the remaining variables were imputed using the multiple imputation method. Continuous variables are presented using medians and quartiles, and categorical variables are presented using frequencies and percentages. Survival probability curves were drawn using the Kaplan-Meier (K-M) analysis, and log-rank tests were used to compare survival probabilities between the trajectory categories. Cox proportional-hazards regression was used to identify differences in mortality risk between patients in different trajectory categories, and results were expressed as hazard ratios (HRs) and 95% confidence intervals (95% CIs). In Model I, only classes were analyzed and no other variables were adjusted. In Model II, age, sex, vital signs, laboratory tests and comorbidities were included in the Cox proportional-hazards regression model and analyzed using forward partial likelihood estimation. We also identified five prespecified subgroups of age, sex, congestive heart failure, cerebrovascular disease, and chronic pulmonary disease. Cox regression analysis was performed using the interaction test to determine whether there was an interaction between the above-mentioned subgroups and the effect of trajectories on in-hospital mortality. Variance inflation factors (VIFs) were used to test for multicollinearity among the independent variables before performing multivariate COX regression.

Probability values of P < 0.05 were considered significant, and all statistical analyses were performed using R software (version 4.2.0).

## Results

Additional file 1: Table S1 listed the VIFs of each covariate, they were all less than 5, indicating that there was no multicollinearity between the variables. The goodness-of-fit statistics of the LGMM for SBP in patients with sepsis are listed in Table 1. AIC, BIC, SABIC, and Entropy all had downward trends from one-class to seven-class models. In the seven-level model, the smaller values of the above four indicators and the Entropy value of > 0.8 indicate that the model had good classification accuracy. In this model, the sample size of the least populated trajectory was 2.82%, which met the predetermined criteria. The posterior probabilities for trajectory classes 1–7 were 89.89%, 91.66%, 91.57%, 87.13%, 81.80%, 87.38%, and 95.84%, respectively, indicating that the model had acceptable accuracy. The seven-class model was therefore the most desirable.

**Table 1:** Statistics for choosing the best number of classes

Number of classes	Log likelihood	AIC	BIC	SABIC	Entropy	%class1	%class2	%class3	%class4	%class5	%class6	%class7
1	− 137,126.8	274,261.6	274,285.7	274,273.0	1.0000000	100.00000						
2	− 130,941.8	261,899.7	261,947.8	261,922.4	0.9256306	70.32173	29.678267					
3	− 128,860.1	257,744.2	257,816.4	257,778.3	0.8875527	46.48720	41.135916	12.376888				
4	− 127,815.1	255,662.3	255,758.6	255,707.8	0.8815714	49.60604	25.640184	16.579120	8.174655			
5	− 127,298.3	254,636.6	254,757.1	254,693.5	0.8763232	44.51740	13.066316	29.448457	9.848982	3.118844		
6	− 126,727.0	253,501.9	253,646.4	253,570.2	0.8715482	10.04596	44.156271	20.091924	12.541037	10.045962	3.118844	
7	− 126,381.4	252,818.9	252,987.5	252,898.5	0.8534338	36.80236	7.452397	8.371635	21.208142	15.200263	8.141825	2.823375

*AIC* Akaike information criterion, *BIC* Bayesian information criteria, *SABIC* sample-adjusted information criteria

**Fig. 1:**
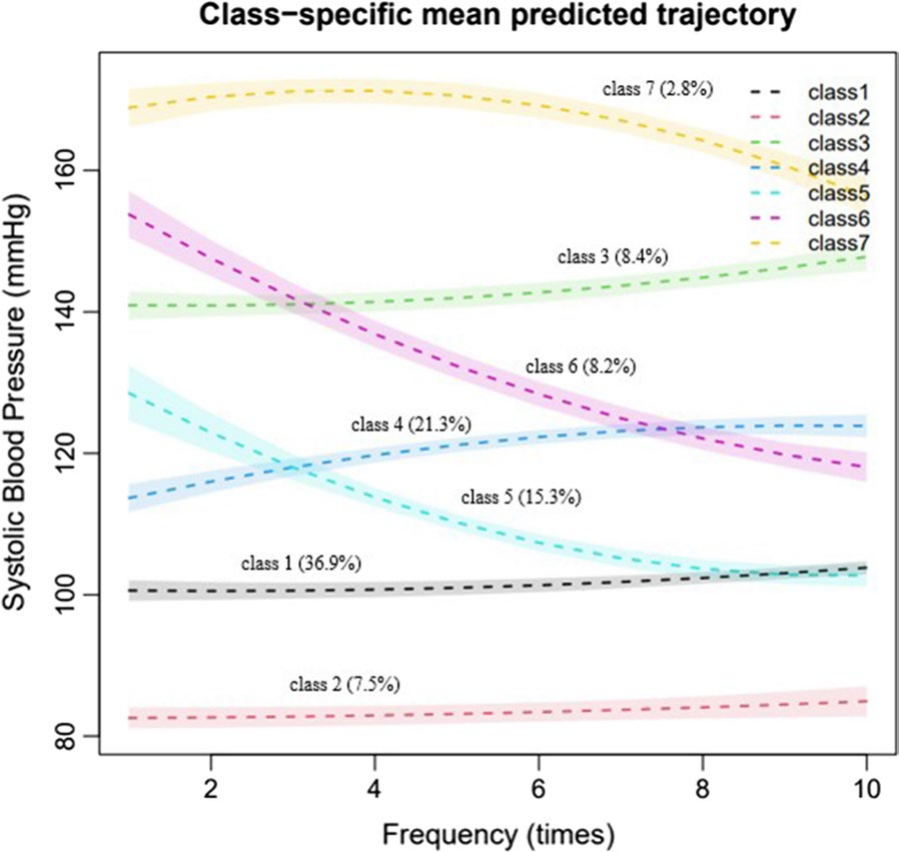
Seven trajectories of the SBP based on LGMM. The shaded area indicates the 95% confdence interval for each trajectory. The percentages in the parentheses indicate the percentages of patients each class accounts for. *SBP* systolic blood pressure, *LGMM* latent growth mixture modeling

The SBP change trajectory of the seven-class model is shown in Fig. 1. The proportion of samples in class 1 was 36.9%, in which SBPs changed steadily with no obvious increasing or decreasing trend, and the mean value was about 100 mmHg. Class 2 accounted for 7.5%, in which the SBP change trend was stable, and the mean value was about 82 mmHg. Class 3 accounted for 8.4%, in which the SBP gradually increased from 140 mmHg, and the change trend was generally stable. Class 4 accounted for 21.3%, in which the SBP had a steady increasing trend, gradually increasing from 110–120 mmHg to 120–130 mmHg. Class 5 accounted for 15.3%, and the SBP had a rapid decreasing trend from about 130 mmHg to 100 mmHg. Class 6 accounted for 8.2%, in which the SBP had a rapid decreasing trend from about 150–160 mmHg to 110–120 mmHg. Class 7 accounted for 2.8%, in which the SBP first had an increasing trend and then a decreasing one. The average SBP value of class 7 exceeded 160 mmHg, and the overall level of class 7 was higher than that of other classes.

The baseline characteristics of patients stratified based on the seven trajectory classes are listed in Table 2. The study included 3034 eligible patients, with males predominating (57%). The median age of the patients was 67 years. The in-hospital mortality rate was 22.3% (N = 676). The in-hospital mortality rates for classes 1 to 7 were 25.5%, 40.5%, 11.8%, 18.3%, 23.5%, 13.8%, and 10.5%, respectively.

**Table 2:** Descriptive characteristics of overall participants and by SBP trajectory

Variables	Overall	Class1	Class2	Class3	Class4	Class5	Class6	Class7	P-value
Number	3034	1121	227	255	646	463	248	86	
Age	67 (56, 77)	66 (55, 77)	66 (57, 78)	69 (57, 78)	68 (57, 79)	67 (56, 77)	67 (56, 78)	66 (56, 76)	0.2
Gender									0.049
Male	1,725 (57%)	648 (58%)	137 (60%)	151 (59%)	364 (57%)	240 (52%)	128 (52%)	57 (66%)	
Female	1,309 (43%)	469 (42%)	90 (40%)	103 (41%)	277 (43%)	223 (48%)	118 (48%)	29 (34%)	
Heart rate (beats/min) (h)	91 (79,104)	93 (79,106)	94 (81, 109)	88 (76, 100)	89 (77, 101)	91 (80, 103)	89 (78, 103)	88 (78, 102)	< 0.001
SBP (mmHg)	108 (101, 118)	105 (99, 112)	98 (90, 105)	121 (111, 135)	112 (105, 122)	108 (102, 115)	114 (106, 125)	125 (113, 145)	< 0.001
DBP (mmHg)	58 (52, 64)	58 (52, 63)	55 (50, 59)	61 (54, 69)	58 (52, 65)	58 (52, 64)	59 (53, 64)	63 (54, 70)	< 0.001
Respiration rate (beats/min)	20.3 (17.6, 23.7)	20.6 (17.7, 24.0)	21.2 (17.9, 25.1)	19.4 (17.0, 22.9)	20.3 (17.5, 23.1)	20.4 (17.8, 23.7)	19.6 (17.2, 23.3)	19.0 (16.7, 22.2)	< 0.001
Temperature (℃) (T)	36.84 (36.48, 37.26)	36.84 (36.44, 37.28)	36.75 (36.20, 37.25)	36.92 (36.60, 37.28)	36.83 (36.49, 37.23)	36.84 (36.50, 37.28)	36.87 (36.58, 37.24)	37.06 (36.57, 37.36)	0.026
SpO2 (%)	97.05 (95.50, 98.50)	96.88 (95.32, 98.34)	96.50 (94.88, 97.96)	97.21 (95.72, 98.62)	97.29 (95.77, 98.59)	96.92 (95.31, 98.53)	97.57 (96.04, 98.71)	97.72 (96.32, 99.13)	< 0.001
AST (U/l)	39 (23, 85)	38 (24, 92)	52 (28, 129)	42 (23, 90)	37 (23, 70)	38 (24, 85)	40 (22, 85)	36 (19, 51)	< 0.001
Albumin (g/dl)	2.90 (2.40, 3.40)	2.90 (2.40, 3.40)	2.80 (2.30, 3.30)	3.30 (2.80, 3.70)	2.80 (2.40, 3.30)	2.80 (2.40, 3.30)	3.20 (2.50, 3.60)	3.40 (3.00, 3.90)	< 0.001
Bicarbonate (mmol/l)	24 (21, 27)	24 (21, 27)	24 (19, 26)	24 (21, 27)	24 (20, 27)	24 (21, 27)	24 (20, 26)	24 (21, 26)	0.3
Neutrophils (%)	80 (68, 87)	81 (69, 88)	79 (63, 87)	78 (63, 85)	80 (68, 87)	79 (65, 86)	84 (71, 90)	86 (78, 91)	< 0.001
PLT (K/µl)	210 (148, 296)	216 (152, 300)	197 (127, 276)	187 (130, 254)	218 (158, 303)	208 (144, 293)	219 (142, 300)	212 (172, 282)	< 0.001
INR	1.30 (1.10, 1.80)	1.30 (1.10, 1.80)	1.50 (1.20, 2.45)	1.20 (1.10, 1.40)	1.20 (1.10, 1.60)	1.40 (1.20, 2.10)	1.20 (1.10, 1.60)	1.25 (1.10, 1.60)	< 0.001
WBC (× 10^9/l)	9 (6, 14)	10 (6, 15)	8 (6, 14)	9 (6, 14)	9 (6, 13)	9 (6, 14)	8 (6, 12)	7 (4, 9)	< 0.001
CK (U/l)	117 (40, 354)	128 (42, 414)	130 (53, 454)	81 (36, 273)	113 (43, 401)	113 (39, 354)	101 (37, 328)	78 (30, 319)	0.042
CK-MB (U/l)	5 (3, 10)	5 (3, 10)	5 (3, 10)	5 (3, 9)	5 (3, 9)	5 (3, 10)	5 (3, 10)	5 (3, 10)	0.4
Creatinine (µmol/l)	1.20 (0.80, 2.10)	1.20 (0.80, 2.10)	1.50 (1.00, 2.95)	1.10 (0.70, 1.80)	1.20 (0.80, 2.10)	1.10 (0.80, 1.85)	1.00 (0.70, 1.60)	1.40 (0.90, 2.80)	< 0.001
Glucose (mg/dl)	126 (98, 166)	114 (95, 151)	130 (100, 172)	137 (106, 190)	137 (101, 193)	110 (83, 146)	134 (102, 183)	288 (162, 550)	< 0.001
Lymphocyte count (× 10^9/l)	11 (6, 19)	10 (6, 18)	13 (9, 22)	13 (8, 24)	10 (6, 18)	12 (7, 21)	9 (5, 18)	7 (4, 11)	< 0.001
Na (mmol/l)	138 (135, 141)	138 (135, 141)	138 (135, 140)	137 (134, 140)	139 (136, 141)	139 (135, 142)	138 (135, 141)	138 (136, 140)	< 0.001
Congestive heart failure, n (%)									0.2
Yes	934 (31%)	358 (32%)	81 (36%)	74 (29%)	201 (31%)	137 (30%)	62 (25%)	21 (24%)	
No	2,100 (69%)	759 (68%)	146 (64%)	180 (71%)	440 (69%)	326 (70%)	184 (75%)	65 (76%)	
Cerebrovascular disease, n (%)									0.009
Yes	290 (9.6%)	88 (7.9%)	13 (5.7%)	27 (11%)	75 (12%)	44 (9.5%)	29 (12%)	14 (16%)	
No	2,744 (90%)	1,029 (92%)	214 (94%)	227 (89%)	566 (88%)	419 (90%)	217 (88%)	72 (84%)	
Chronic pulmonary disease, n (%)									0.5
Yes	843 (28%)	314 (28%)	65 (29%)	78 (31%)	182 (28%)	124 (27%)	55 (22%)	25 (29%)	
No	2,191 (72%)	803 (72%)	162 (71%)	176 (69%)	459 (72%)	339 (73%)	191 (78%)	61 (71%)	

**Fig. 2:**
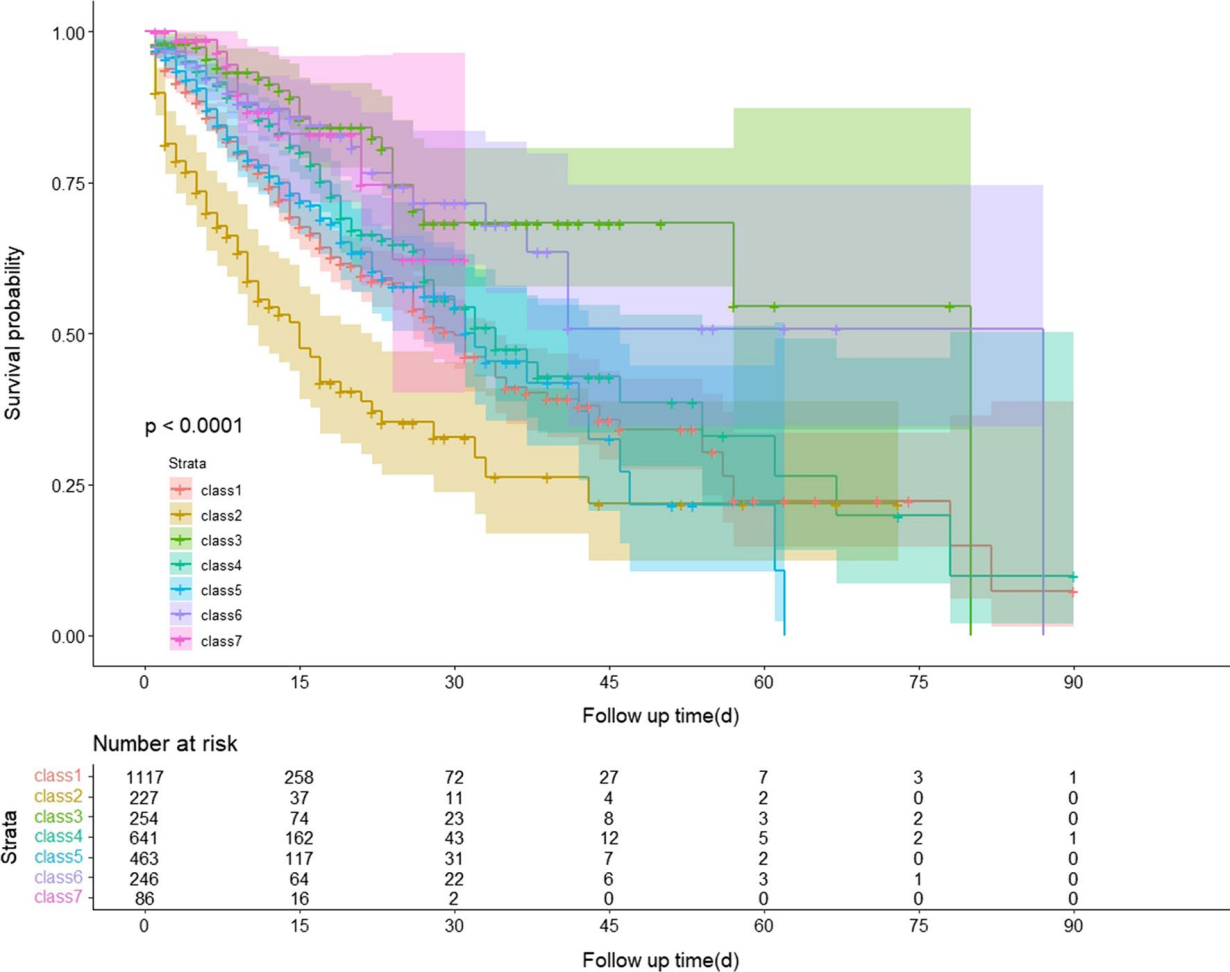
Survival probability curve during hospitalization of patients with different trajectories of the SBP

The survival probabilities of each class plotted using the K-M analysis are shown in Fig. 2. Patients in class 2 had the highest mortality rate, and the results of the log-rank test indicated that the survival probability differed significantly between the classes (P < 0.001).

The results of the Cox proportional-hazards regression analysis are listed in Table 3. In the univariate analysis (Model I), with class 1 as a reference, the HR (95% CI) values of classes 2 to 7 were 1.925 (1.522–2.432), 0.394 (0.271–0.575), 0.698 (0.563–0.866), 0.939 (0.753–1.172), 0.494 (0.346–0.705), and 0.467 (0.240–0.907), respectively. In the multivariate analysis (Model II), with class 1 as a reference, the HR (95% CI) values of classes 2 to 7 were 1.930 (1.515–2.459), 0.356 (0.241–0.527), 0.662 (0.533–0.823), 0.879 (0.703–1.098), 0.618 (0.432–0.885), and 0.677 (0.346–1.324), respectively. Both models suggest that when the SBP change trend of patients with sepsis conforms to class 2, the in-hospital mortality risk is the highest (P < 0.05), and when it conforms to class 3, the in-hospital mortality risk is the lowest (P = 0.009).

**Table 3:** Results of Cox proportional hazard models

Class	Model I		Model II	
	HR (95% CI)	P value	HR (95% CI)	P value
Class 1	Reference		Reference	
Class 2	1.925 (1.522–2.432)	< 0.001	1.914 (1.503–2.437)	< 0.001
Class 3	0.394 (0.271–0.575)	< 0.001	0.338 (0.228–0.502)	< 0.001
Class 4	0.698 (0.563–0.866)	0.001	0.677 (0.545–0.841)	< 0.001
Class 5	0.939 (0.753–1.172)	0.579	0.897 (0.718–1.121)	0.340
Class 6	0.494 (0.346–0.705)	< 0.001	0.614 (0.428–0.879)	0.008
Class 7	0.467 (0.240–0.907)	0.025	0.668 (0.341–1.309)	0.240

**Fig. 3:**
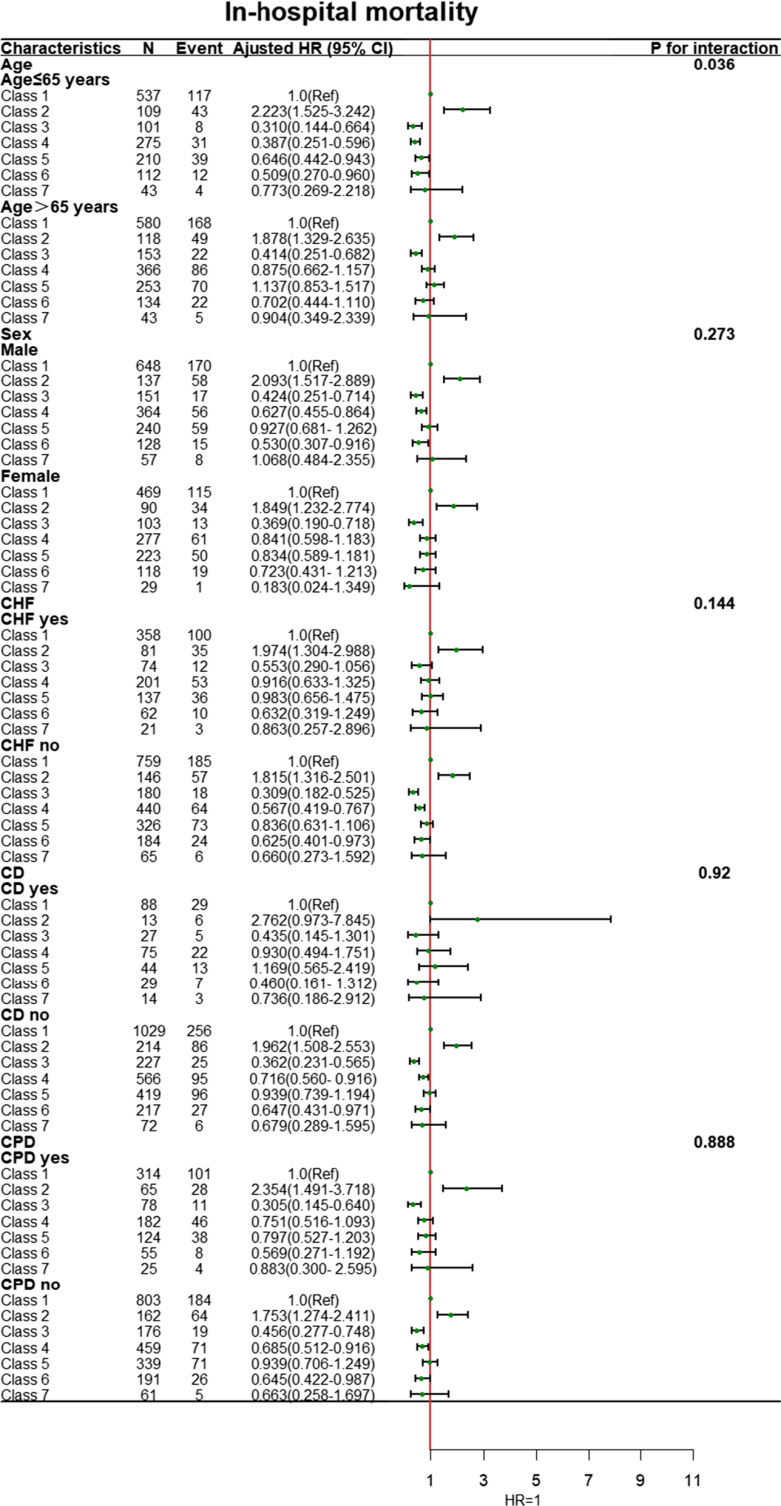
Prespecified subgroup analyses

The study also performed subgroup analyses for the following variables: age, sex, congestive heart failure, chronic lung disease, and cerebrovascular disease. The results indicated that in the subgroups of sex, congestive heart failure, cerebrovascular disease, and chronic pulmonary disease, the above-four variables and SBP trajectories did not interact with in-hospital mortality (P > 0.05). A nominal interaction occurred between age and SBP trajectory in in-hospital mortality (P < 0.05). Figure 3 presents the subgroup analysis results.

## Discussion

Sepsis is one of the most common causes of death in ICUs. The latest cross-sectional survey on the epidemiological characteristics of ICUs in China revealed that the incidence of sepsis in ICUs was 23.9%, and that the 90-day mortality rate was 35.5% [10]. There are about 1.5 million patients with sepsis in the United States every year, and the medical expenses for sepsis treatment are as high as 24 billion dollars, with an increasing trend each year [11]. Early monitoring of the changes in patient conditions and timely intervention are highly significant for the prognosis of patients with sepsis. SBP is one of the readily available indicators in the ICU that can be used to quickly and accurately identify changes in the vital signs of a patient. Blood pressure is affected by myocardial contractility, blood volume, and vascular tension. Changes in blood pressure can indirectly reflect changes in these three factors. It is therefore very important to dynamically monitor the blood pressure trajectory.

The present study used LGMM to conduct trajectory analysis and to establish a model of ten SBP values measured after admission in patients with sepsis, and to explore the relationship between trajectory class and the in-hospital mortality risk of these patients. The findings indicated that in both Model I and Model II, class 2 with the lowest SBP had the highest mortality risk. In the multivariate analysis, class 3 had the lowest mortality risk, which constitutes the most valuable finding of this study. The SBP trajectory in class 3 can be used as a blood pressure management target for patients with sepsis within 10 h of admission to provide a reference for physicians. We observed a sharp drop in SBP in class 6, and Cox proportional-hazards regression analysis indicated that class 6 had a better prognosis than class 2, suggesting that the prognosis is worse for a persistent hypotensive state than for a large decrease in SBP, which can help clinicians to identify high-risk patients as early as possible and thereby provide timely treatment. Both class 3 and class 4 had increasing trends, and Cox proportional-hazards regression analysis indicated that class 3 and class 4 had better prognoses than did class 2. Class 1 had a stable trend, with a SBP of about 100 mmHg, and its prognosis was also better than that of class 2. The difference between class 5 and the reference class was not significant (P > 0.05).

A study by Strandgaard S on the physiology of chronic hypertension found that organ pressure-flow autoregulation shifts to the right in patients with high blood pressure, which may improve organ perfusion and ultimately improve survival, thereby possibly justifying an elevation in the blood pressure [12]. However, excessive SBP increases the cardiac pressure load and leads to glomerular hyperperfusion, which is a good explanation for the risk of death being lower in class 3 (140 mmHg) than in class 7 (160 mmHg). Worsening microcirculatory changes is a cause of organ failure and death in patients with sepsis [13]. From a pathophysiological perspective, in patients with sepsis, decreased vascular tone leads to relative hypovolemia, and vascular leakage leads to absolute hypovolemia [14]. Jhanji et al. found that in patients with hypotensive sepsis, the use of escalating doses of norepinephrine to achieve higher mean arterial pressure (MAP) helped to improve systemic hemodynamics and tissue oxygenation delivery, and did not exacerbate the abnormality of microcirculatory blood flow [15]. In addition, in order to correct hypovolemia and improve tissue perfusion, the guidelines recommend that crystalloids should be administered intravenously at a level of least 30 mg/kg within the first 3 h after admission to increase blood volume, and norepinephrine should be administered at the same time to increase blood pressure [16]. However, excessive fluid resuscitation was independently associated with increased sepsis mortality [17]. We therefore believe that when supplementing the blood volume, more attention should be paid to changes in blood pressure and increased blood pressure over time. Some literature recommends that the MAP of patients with sepsis should not be lower than 65 mmHg [1]. Many previous authors believed that the MAP of patients with sepsis being continuously lower than 65 mmHg will lead to a poor prognosis, and so MAP should be increased in time to improve the prognosis. The findings of Lee et al. also suggest that increasing MAP from 65 mmHg by 5–10 mmHg may improve the prognosis [18], but there is no evidence that higher MAP is associated with improved survival probability [19].

There have been few studies on SBP and the in-hospital mortality risk in sepsis, and our study of SBP trajectory also corroborates the findings of Lee et al. Patients in class 2 had the lowest SBP and the highest mortality risk, class 7 had higher SBP values than class 3, but class 7 had a higher mortality risk than class 3, suggesting that higher SBP does not necessarily decrease the risk of mortality in patients with sepsis. Higher blood pressure values in class 7 increased the resistance during myocardial contraction and lead to hyperperfusion of the brain and glomeruli, which increases the risks of renal insufficiency and cerebral edema. One of the main reasons for class 2 having the highest mortality risk was that low SBP leads to insufficient organ and tissue perfusion and cell metabolism dysfunction, which result in damage to important organs such as the heart and brain [20–22]. Timely monitoring and intervention of SBP changes is therefore one of the important measures in sepsis treatment [22, 23]. Our study showed that SBP in class 3 (approximately 140 mmHg) were associated with a lower risk of in-hospital mortality. Therefore, we recommend that patients with sepsis maintain SBP at 140 mmHg after admission to reduce the risk of in-hospital mortality. However, this is a single center retrospective study, we hope to conduct randomized controlled trials in the future to obtain more information.

We performed subgroup analyses for age, sex, congestive heart failure, cerebrovascular disease, and chronic pulmonary disease, with the results indicating that age was a predictor of in-hospital mortality. Patients aged < 65 years in class 2 had a higher mortality risk than did patients aged > 65 years. A previous study found that patients with sepsis aged > 60 years had a higher mortality risk than did younger adults [24]. The results of our study were inconsistent with previous reports. The elderly may have comorbidities such as decreased immunity, diabetes, and hypertension [25]. When microcirculation disorders or organ insufficiency occur in the elderly, their compensatory ability is low, and so they have a worse prognosis than young people. However, in patients with sepsis, young people have a stronger compensatory ability, which may cause a more-severe systemic inflammatory response syndrome, and at the same time a large number of inflammatory factors are released, causing severe multiple organ dysfunction; this mechanism may explain why the mortality risk is higher in young patients than in the elderly.

This study had some limitations. First of all, SBP values were affected by many factors, such as the use of vasopressors before entering the ICU and a hypertension history. These factors were not included in this study, which was due to the lack of some information in the MIMIC-III database. Whether variables not included in the study affect the prognosis of patients with sepsis needs to be further explored in follow-up studies. We hope that fewer or more SBP measurements will be analyzed in future studies. In addition, patients who died without completing 10 SBP measurements were excluded from our study, which may have contributed to survivorship bias. We hope that in future studies, the number of SBP measurements during the same observation period will not be limited, and patients who died during the observation period will be included in the study to avoid survivorship bias. Furthermore, we only investigated the impact of SBP trajectory on the prognosis of patients with sepsis, and so future studies should combine the trajectory of other variables to determine their impacts on prognosis.

## Conclusion

The SBP trajectory within 10 h of admission in patients with sepsis has an impact on in-hospital mortality. Patients with persistently low or high SBP or with large decreases in SBP had a higher mortality risk than when SBP was maintained at about 140 mmHg. The trend of SBP in patients should therefore be closely observed, and corresponding treatment should be provided as soon as possible to improve their prognoses.

## Supplementary Information

**Additional file 1**: **Table S1**. Variance inflation factor for in-hospital mortality.

## Data Availability

The MIMIC-III data are available at https://mimic.mit.edu/docs/iii/.

## Abbreviations

MIMIC III: Medical Information Mart for Intensive Care III
AST: Aspartate aminotransferase
CK: Creatine kinase
CK-MB: Creatine kinase myocardial band
PLT: Platelets
SBP: Systolic blood pressure
DBP: Diastolic blood pressure
Spo2: Blood oxygen saturation
Na: Serum sodium
CHF: Congestive heart failure
CD: Cerebrovascular disease
CPD: Chronic pulmonary disease
