# Supplementary material for: Influence of systolic blood pressure trajectory on in-hospital mortality in patients with sepsis
Source: BMC Infect Dis. 2023 Feb 13;23:90. doi: 10.1186/s12879-023-08054-w (PMC9926677; doi:10.1186/s12879-023-08054-w)
Supplement: Supplementary file 1 — Additional file 1: Table S1. Variance inflation factor for in-hospital mortality. [file 12879_2023_8054_MOESM1_ESM.docx]

Table S1. Variance inflation factor for each variable

| Variable | GVIF | Df | GVIF^(1/(2*Df)) |
| --- | --- | --- | --- |
| Age | 1.245703 | 1 | 1.116111 |
| Gender | 1.088488 | 1 | 1.043306 |
| Male | 1.126049 | 1 | 1.061155 |
| Female | 1.536846 | 1 | 1.239696 |
| Heart rate | 1.526875 | 1 | 1.235668 |
| SBP | 1.892244 | 1 | 1.375589 |
| DBP | 1.659252 | 1 | 1.288120 |
| Respiration rate | 1.329241 | 1 | 1.152927 |
| Temperature | 1.296398 | 1 | 1.138595 |
| SpO2 | 1.223009 | 1 | 1.105897 |
| AST | 1.251706 | 1 | 1.118797 |
| Albumin | 1.232273 | 1 | 1.110078 |
| Bicarbonate | 1.315450 | 1 | 1.146931 |
| Neutrophils | 3.010166 | 1 | 1.734983 |
| PLT | 1.186420 | 1 | 1.089229 |
| INR | 1.215825 | 1 | 1.102645 |
| WBC | 1.091461 | 1 | 1.044730 |
| CK | 1.291102 | 1 | 1.136267 |
| CK-MB | 1.454500 | 1 | 1.206027 |
| Creatinine | 1.536284 | 1 | 1.239469 |
| Glucose | 1.437665 | 1 | 1.199027 |
| Lymphocyte count | 2.910225 | 1 | 1.705938 |
| Na | 1.128045 | 1 | 1.062095 |
| Congestive heart failure | 1.232851 | 1 | 1.110338 |
| Cerebrovascular disease | 1.052175 | 1 | 1.025756 |
| Chronic pulmonary disease | 1.082466 | 1 | 1.040416 |

**Table legends**

Table S1: Variance inflation factor for each variable
